# Integrating Nutrition into Mental Health Care: An Exploratory Cross-Sectional Survey of Training, Practices, Barriers, and Collaboration Among Mental Health and Nutrition Professionals

**DOI:** 10.3390/nu18162601

**Published:** 2026-08-08

**Authors:** Riccardo Gurrieri, Vladimir Hedrih, Ephi Morphew-Lu

**Affiliations:** 1 The Center for Nutritional Psychology, 467 Saratoga Ave, No 173, San Jose, CA 95129, USA; ephilu@nutritional-psychology.org; 2 Health Science Interdisciplinary Center, Sant’Anna School of Advanced Studies, 56127 Pisa, Italy; 3 Faculty of Philosophy, University of Niš, Ćirila i Metodija 2, 18000 Niš, Serbia; vladimir.hedrih@filfak.ni.ac.rs

**Keywords:** nutritional psychology, interdisciplinary collaborations, nutritionist/dietitians, psychologists, perceived barriers, integrated care

## Abstract

**Background/Objectives**: The integration of nutrition into the psychological sciences and mental healthcare has gained increasing attention with the emergence of Nutritional Psychology as an interdisciplinary field. However, little is known about how professionals approach this interface or the barriers they face. This study aimed to explore the extent to which mental health and nutrition professionals engage with each other’s domains. **Methods**: This cross-sectional study analyzed survey responses from 196 professionals, including mental health professionals, nutrition professionals, and individuals from related fields recruited through the individual and institutional professional network of the Center for Nutritional Psychology, San Jose, CA, USA. The goal was to examine training, practices, perceived barriers, and interest in collaboration between these professions. Descriptive statistics summarized categorical and quantitative variables. Mann–Whitney U tests with rank-biserial correlation examined ordinal data. Exploratory factor analysis (principal axis factoring, varimax rotation) was applied to barrier ratings. **Results**: A total of 78.3% of participating mental health professionals reported addressing nutrition in their practice at least occasionally, while a similar percentage (78.5%) of participating nutrition professionals discussed mental health with clients at least occasionally. Despite this engagement, 25% of all participating nutrition professionals and 51% of all participating mental health professionals reported no formal training in the complementary field, with most of their knowledge acquired informally through self-study or workshops. A total of 11 mental health professionals and 13 nutrition professionals did not answer the question about formal education in the complementary field. The highest-rated perceived barriers included a lack of training, unclear professional boundaries, and a lack of a common language, whereas a lack of evidence was perceived as the least critical. Importantly, 99% of respondents who answered the question (*n* = 84, while 112 participants did not respond), or 42% of all participants, expressed interest in evidence-based interdisciplinary training. A total of 96% of those who answered the question (*n* = 133, while 63 participants did not answer) or 65% of all participants agreed that collaboration would improve patient outcomes. However, only 49% of all participating nutrition professionals and 50% of all participating mental health professionals reported having collaborative experience. A total of 7 mental health professionals and 16 nutrition professionals did not answer the question about collaborative experiences. **Conclusions**: Within this self-selected sample, respondents reported frequent cross-disciplinary engagement, limited formal training, and interest in interdisciplinary education and collaboration. These exploratory findings require confirmation in independently recruited and better-characterized samples.

## 1. Introduction

The relationship between dietary intake and mental health is increasingly recognized as bidirectional and clinically relevant [1,2,3,4]. A growing body of research has highlighted the possible influence of nutrition on psychological processes, brain function, and psychopathological outcomes, including mood, cognition, behavior, and emotional regulation [5,6,7,8,9,10,11,12]. In parallel, the emergence of Nutritional Psychology as an emerging interdisciplinary field aims to systematize these findings, provide a shared theoretical framework, and create novel language, tools, and training to support the integration of nutritional science into clinical mental health practice [13].

Despite this momentum, mental health and nutrition professionals often lack adequate training to implement such an integrated approach within scope of practice. Surveys show that between 47.5% and 66.3% of psychologists report no formal nutrition education, yet many still address dietary issues in therapy sessions [14,15]. Similarly, dietitians and nutritionists report some exposure to psychological content, often limited to basic undergraduate coursework, and feel underprepared to address mental health concerns within their scope of practice [14,15,16,17]. Nevertheless, interest in additional training remains high across both disciplines; up to 97% of respondents in recent studies expressed a desire to expand their knowledge of nutrition-mental health interconnections [14,15].

Beyond individual diet–brain relationships, cross-cultural and biopsychosocial factors significantly modulate how nutrition interfaces with mental health. Cultural variation in genetic polymorphisms, self-construal, and metabolic profiles has been linked to differences in depression prevalence and antidepressant response [18,19,20]. Moreover, cultural norms and socioeconomic conditions influence the relationship between body mass index (BMI) and depression, with positive correlations found in Western samples but often inverse correlations found in East Asian populations [21,22]. Such findings highlight that the interplay between diet, biology, and mental health cannot be understood apart from its cultural and ecological context.

While promising models of interprofessional collaboration have emerged, such as Interprofessional Enhanced Cognitive Behavioral Therapy (CBT-IE) in the treatment of eating disorders [23], the systematic implementation of Nutritional Psychology within broader mental health practice remains underdeveloped. This gap is especially relevant for psychological domains such as mood, anxiety, emotion regulation, stress responsiveness, eating-related behaviors, and overall psychological well-being, where nutrition-related factors may interact with cognitive, emotional, behavioral, and relational processes [16]. Similar gaps have also been observed in obesity and weight management, where psychological interventions such as Cognitive Behavioral Therapy (CBT), Motivational Interviewing (MI), and Acceptance and Commitment Therapy (ACT) remain inconsistently implemented in community and Tier 2 care services, highlighting the need for systematic integration of essential evidence-based psychological approaches into primary care [24]. While mental health practitioners focus primarily on emotional, behavioral, and cognitive processes, nutrition professionals emphasize dietary assessment and metabolic health. Barriers to this integration include a lack of formal interdisciplinary education, limited role clarity, absence of a shared clinical language, and fears of overstepping professional boundaries [16,17,25]. Additional obstacles concern economic and administrative aspects, such as the lack of reimbursement mechanisms or billing codes for interdisciplinary services, limited insurance coverage, and the resulting financial inaccessibility of integrated care models [17,26].

Evidence directly comparing mental health and nutrition professionals within the same survey across cross-disciplinary training, professional practices, perceived barriers, and collaborative experiences remains limited. To address these gaps, it is essential to better understand how professionals from both fields currently approach the nutrition–mental health interface, how they perceive collaboration, and what barriers and training needs they identify. The development of evidence-based educational programs and collaborative frameworks requires input from practitioners working at this intersection. Without this information, integration efforts may remain fragmented and lack ecological validity.

The present study aims to:

- Explore how much mental health and nutrition professionals currently engage with each other’s domains in clinical practice;
- Determine the nature and extent of formal and informal training among mental health and nutrition professionals in relationships between nutrition and mental health;
- Identify perceived barriers and training gaps;
- Assess interest in interdisciplinary collaboration, education, and training.

Through a structured online survey, we examined: (1) the frequency with which mental health professionals address nutrition and conversely, nutrition professionals address mental health, (2) the characteristics of their formal and informal training in areas linking nutrition and mental health, (3) their openness to evidence-based interdisciplinary education, and (4) their actual experiences with interprofessional collaboration. This study seeks to inform the development of practical frameworks and training programs to inform the future development and evaluation of scope-appropriate interdisciplinary education and collaborative care at the nutrition-mental health interface.

## 2. Materials and Methods

This study employed a cross-sectional, exploratory design based on an online survey conducted by the Center for Nutritional Psychology, San Jose, CA, USA (CNP) in 2026. The survey was designed to explore current practices, training, perceived barriers, and attitudes toward interprofessional collaboration between mental health and nutrition professionals. Item generation was informed by a review of existing surveys exploring nutrition–mental health integration [14,15,16,17,21].

The survey comprised five sections addressing key domains of professional engagement at the interface between nutrition and mental health. It collected information on respondents’ professional background, cross-disciplinary practice (frequency of addressing nutrition or mental health), training (type and extent of formal and informal education in the complementary field), perceived barriers to integration, and collaborative experience and interest. The questionnaire was developed collaboratively by a multidisciplinary team including psychologists, mental health professionals, and dietitian/nutrition professionals affiliated with CNP [25]. It included items assessing professional background, frequency of addressing nutrition (among mental health professionals) or mental health (among nutrition professionals) in clinical practice, formal and informal training in nutrition related to mental health, perceived barriers to integration, interest in interdisciplinary training, and perceptions regarding the clinical utility of collaboration.

Participants’ responses on formal and informal education were used to estimate the quantity of nutrition-related training in mental health they had received. For formal training, points were assigned as follows: 1 point for one undergraduate course, 2 points for a professional certificate, 3 points for more than one undergraduate course, and 4 points for graduate coursework. Informal training was operationalized as the number of different informal learning sources reported by each participant, including self-directed learning, workshops or similar activities, and professional activities or mentoring. This variable reflects the diversity of reported learning sources rather than the duration, intensity, or quality of informal education.

**Figure 1:**
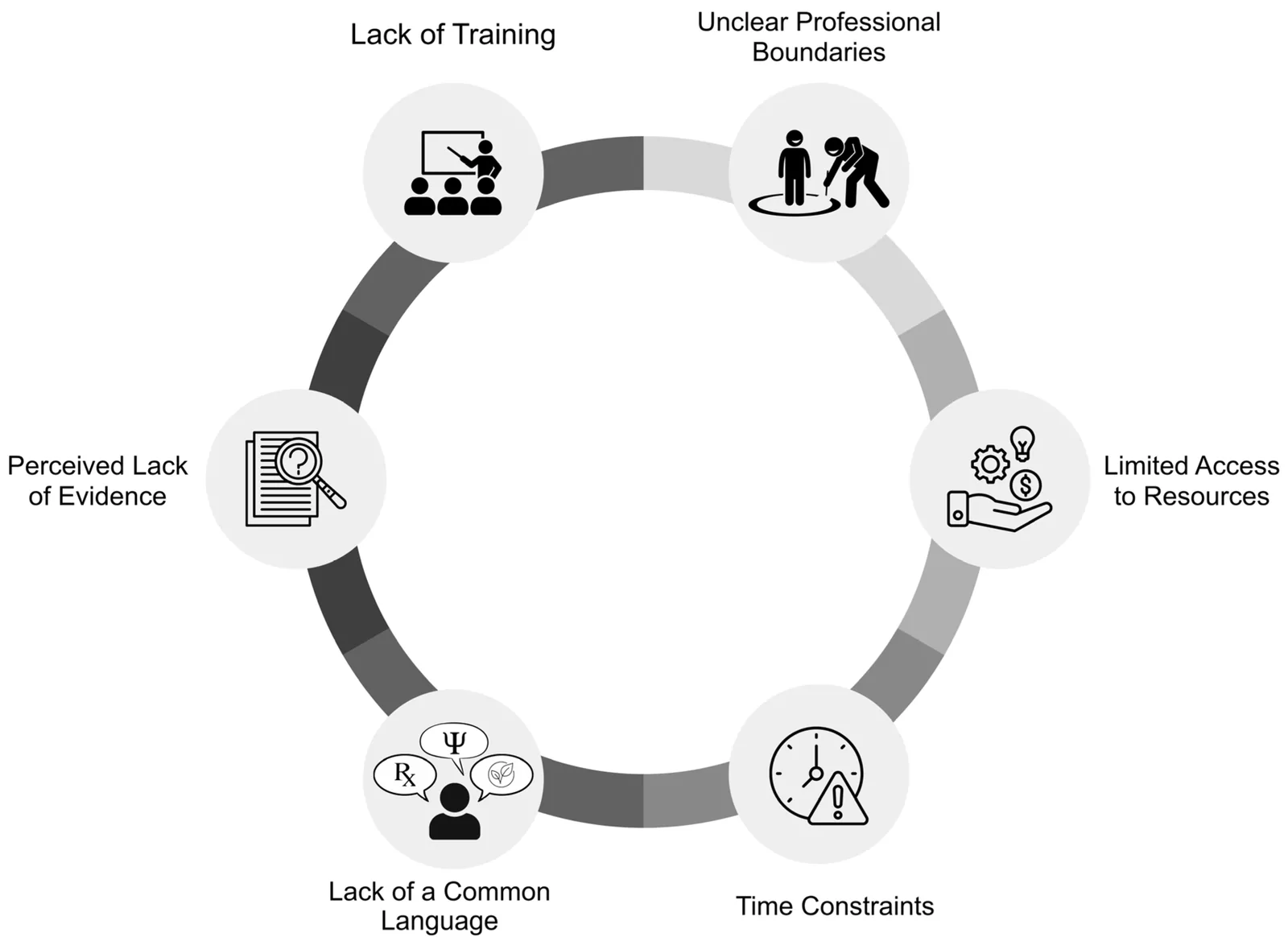
The six examined perceived barriers to integration of nutrition knowledge in mental health work and of mental health knowledge in nutrition practice.

Respondents were also asked to rate six common barriers to integration (lack of training, unclear professional boundaries, limited access to resources, time constraints, lack of a common language, and perceived lack of evidence) on a scale of 1 (No barrier) to 5 (A very large barrier) (see Figure 1).

An initial version of this survey was used in a previous study that was not published, but was used to improve the formulation of the survey questions and answer options.

A convenience sampling approach was used. The survey was disseminated between February and May 2026 via professional mailing lists, social media channels, institutional networks, and direct outreach to clinicians affiliated with CNP and collaborating institutions. Eligibility criteria included being a mental health or nutrition professional or trainee working or training in a relevant field (e.g., psychology, psychiatry, counseling, dietetics, nutrition science, wellness coaching or related profession). Respondents self-identified their professional background, and those who could not be classified as mental health or nutrition professionals were included in a heterogeneous “Other” category comprising students, educators, chefs, a social worker, a wellness coach, a physical therapist, and other occupations. Participation was voluntary and anonymous, though respondents could optionally provide their email address to receive follow-up communications (in which case the survey became confidential). Demographic and professional information collected included the participants’ professional category or title (e.g., psychologist, dietitian, counselor, and educator). However, no additional variables such as years of professional experience, licensure status, geographic location, type of patient population served, or primary practice setting (e.g., private vs. public sector) were collected.

Frequencies and percentages were calculated for categorical variables, and means, medians, and standard deviations were calculated for quantitative variables. The Mann–Whitney U test was used for comparing mean ranks of variables that could be considered ordinal, while the rank-biserial correlation coefficient (r_r_bis) was used as a measure of effect size. Exploratory factor analysis using the principal axis factoring method and varimax orthogonal rotation was used to explore the nature of associations between barrier ratings.

Analyses were performed using Microsoft Excel, JASP (version 0.98.1), and IBM SPSS Statistics, Version 25.0 (IBM Corp., Armonk, NY, USA).

## 3. Results

**Figure 2:**
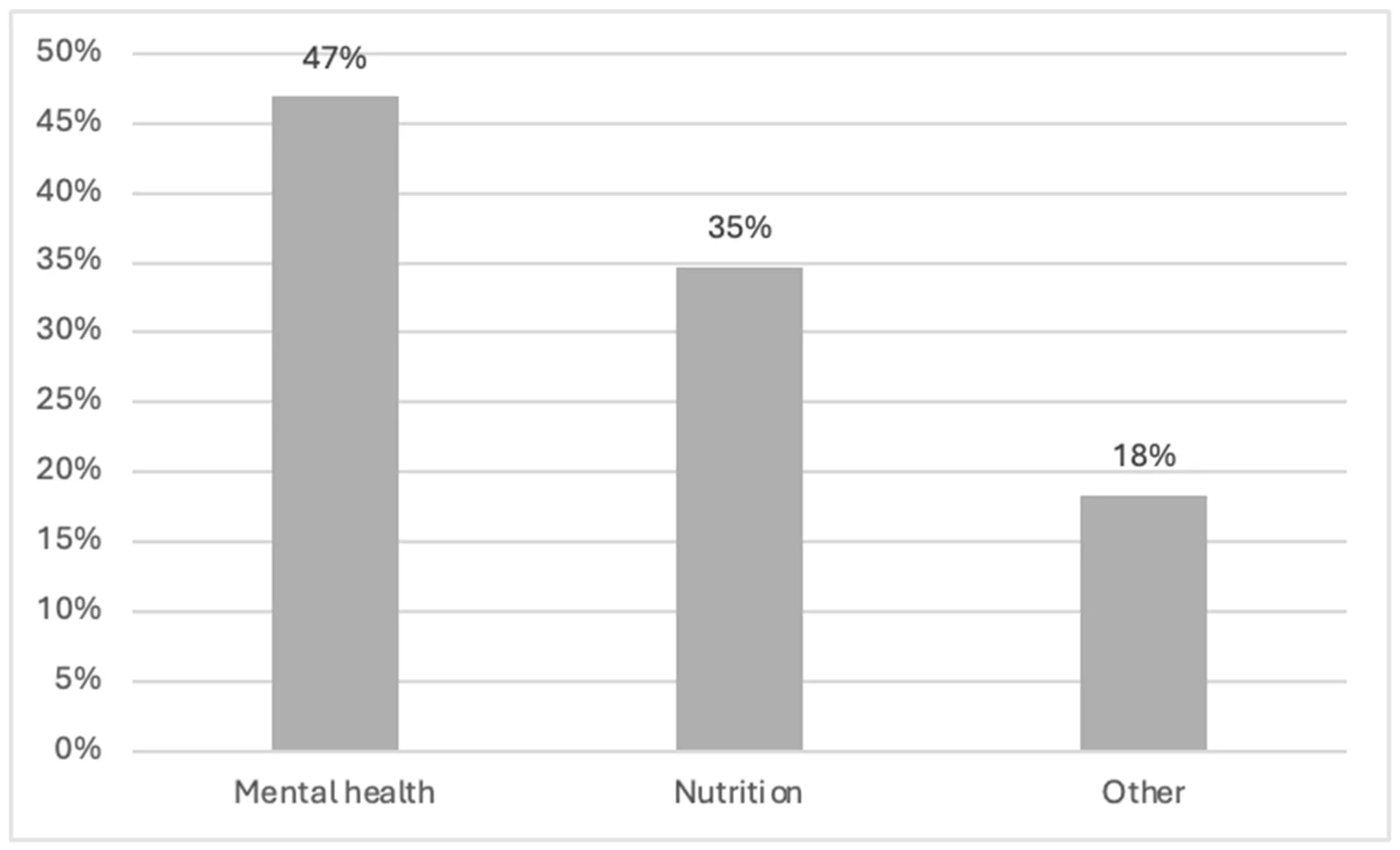
Professional Roles of Participants (*n* = 196).

A total of 196 professionals completed the survey (Figure 2). Of these, 46.9% (*n* = 92) were mental health professionals, 33.2% (*n* = 65) were nutrition professionals, and 19.9% (*n* = 39) belonged to other professions. The mental health professionals category included psychologists and counselors across various specializations. The nutrition specialists were most often dietitians/nutritionists. The “Other” professionals group consisted mainly of educators and students, but also included 3 chefs, a social worker, and individuals with several other professions.

### 3.1. How Often Do Psychologists Address Nutrition, and Dietitians Address Mental Health?

**Figure 3:**
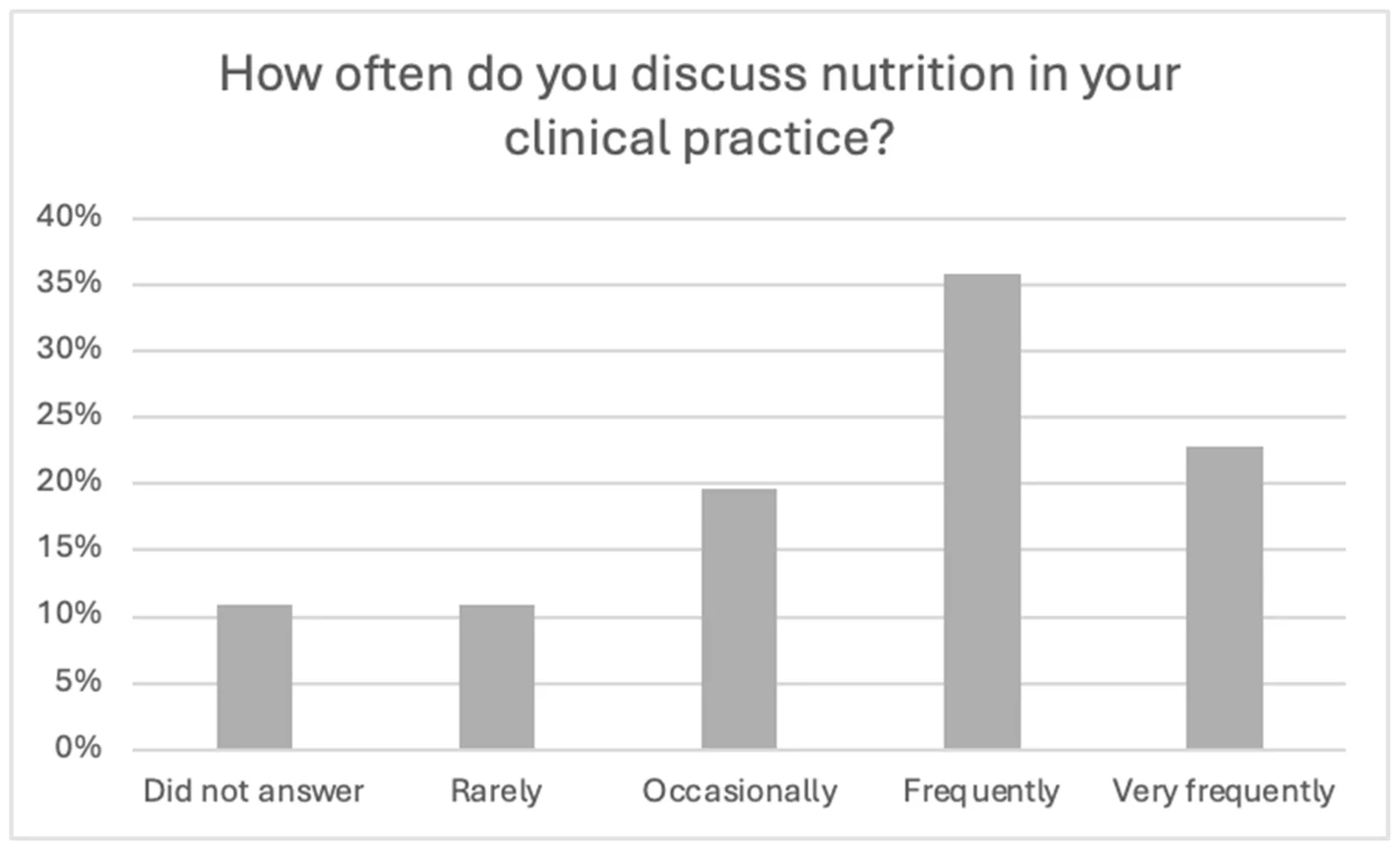
Frequency of Nutrition Discussions in Mental Healthcare (*n* = 92).

Among participants who identified as psychologists or other mental health professionals (*n* = 92), 22.8% (*n* = 21) of all participating mental health professionals reported discussing nutrition very frequently in their clinical practice, and 35.9% (*n* = 33) reported doing so frequently. An additional 19.6% (*n* = 18) reported addressing nutrition occasionally, while 10.9% (*n* = 10) did so rarely. Zero participants reported never discussing nutrition in their sessions, but 10 mental health professionals (10.9%) did not answer this question. These findings indicate that the vast majority of mental health professionals surveyed do address nutrition to some extent in clinical settings. Results are illustrated in Figure 3.

**Figure 4:**
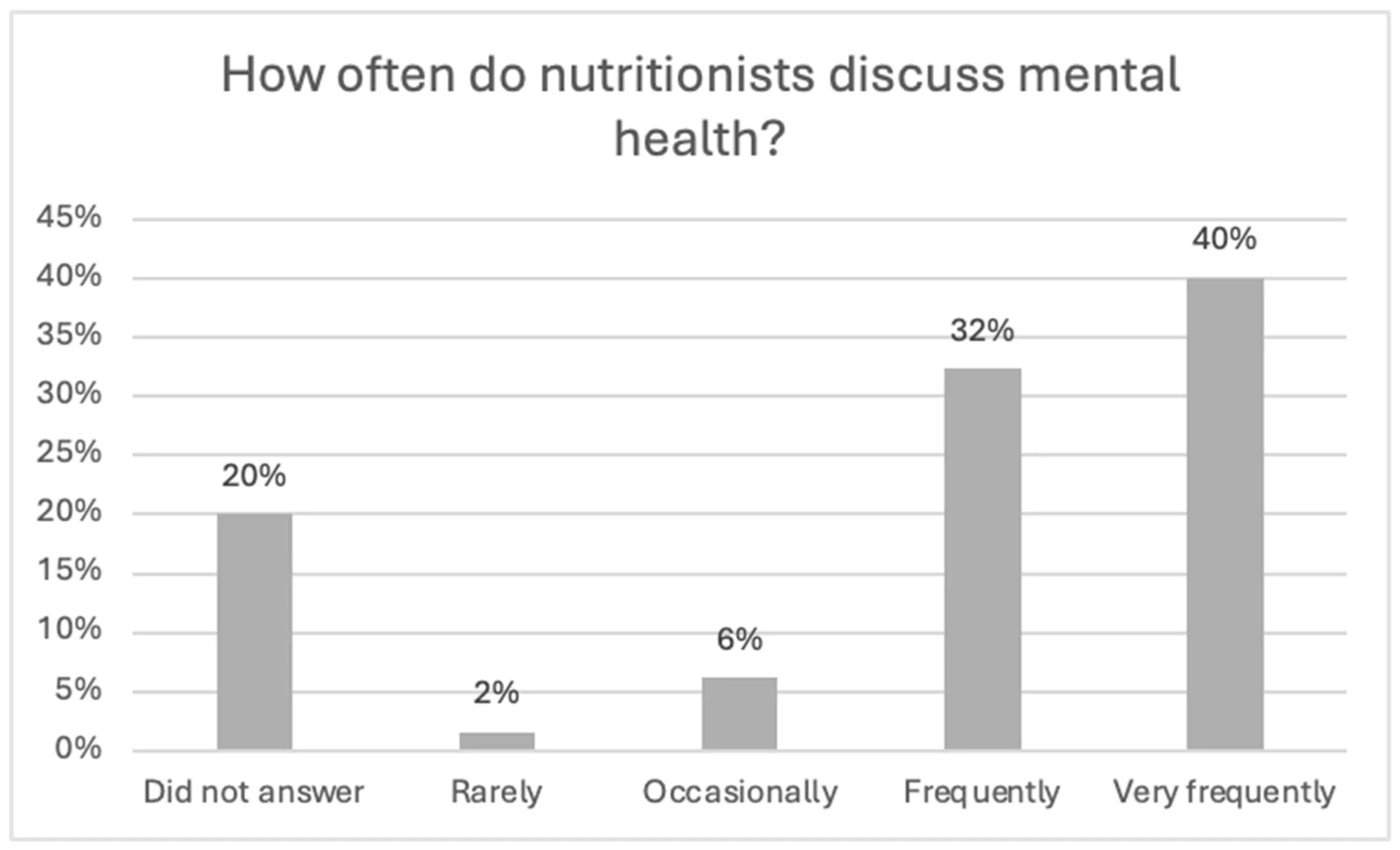
Mental Health Discussions in Clinical Practice: Dietitians & Nutritionists (*n* = 65).

Conversely, among respondents identifying as dietitians or nutritionists (*n* = 65), the majority reported routinely addressing mental health in their clinical work. Specifically, 26 participants (40% of all participating nutrition professionals) stated they do so very frequently, while 21 (32.3%) indicated they do so frequently. Only a small number of participants reported addressing mental health occasionally (4; 6.2%) or rarely (1; 1.5%). A total of 13 nutrition professionals (20.0%) did not answer this question. These data suggest that among nutrition professionals who do discuss mental health, most do so frequently or very frequently (72.3%). Results are presented in Figure 4.

If the reported frequencies are treated as ordinal values and central tendencies of how often mental health professionals address nutrition compared with how often nutrition specialists address mental health issues, results showed that nutrition specialists report addressing mental health issues (M = 3.385, SD = 0.718) slightly more frequently than mental health professionals report addressing nutrition (M = 2.793, SD = 0.965). The result of the Mann–Whitney U test comparing mean ranks of these two groups is statistically significant, and the effect size indicates a small difference (N1 = 82, N2 = 52, U = 1389.5, z = −3.601, *p* < 0.001, r_r_bis = 0.348).

### 3.2. Formal and Informal Training and Interest in Nutrition Related to Mental Health

Among respondents who were mental health professionals (*n* = 92), 51% (*n* = 47) reported having received no formal training. The most frequently reported forms were professional certificates or training programs (25% of all participating mental health professionals or 28% of mental health professionals who answered the questions; *n* = 23), followed by graduate-level coursework (8% of all participating mental health professionals, or 9% of mental health professionals who answered the question; *n* = 7). A smaller proportion reported having completed more than one undergraduate course (5% of all participating mental health professionals or 6% of the mental health professionals who answered the question; *n* = 5) or a single undergraduate course (4% of all participating mental health professionals or 5% of mental health professionals who answered the question; *n* = 4). Several participants reported completing more than one form of formal education. A total of 11 mental health professionals did not answer the question about formal education, while 10 did not respond to the question about informal straining. Overall, while some mental health professionals reported exposure to formal educational pathways, these findings underscore the persistence of a significant gap in structured nutrition training among those working in mental health fields.

Of the 65 nutrition professionals, 16 (25% of all participating nutrition professionals) reported having no formal education in mental health. A total of 13 did not answer this question Professional certificates or training programs were the most common form of education in mental health in this group (34% of all participating nutrition professionals or 42% of nutrition professionals who answered this question, *n* = 22), followed by more than one course (23% of all participating nutrition professionals or 29% of the nutrition professionals who answered the question, *n* = 15), one undergraduate course (12% of all participating nutrition professionals or 15% of the nutrition professionals who answered the question, *n* = 8), and graduate coursework (11% of all participating nutrition professionals or 14% of those who answered the question, *n* = 7). Participants often reported having more than one form of formal education in mental health related to nutrition.

A total of 11 mental health professionals and 13 nutrition professionals did not answer the question about formal education in the complementary field. A total of 10 mental health professionals and 13 nutrition professionals did not answer the question about informal education in the complementary field.

Comparing mental health professionals and participants whose primary area of work is in the field of nutrition on the quantity of education in nutrition related to mental health versus mental health related to nutrition results show that nutrition professionals tend to report more formal education in nutrition than mental health professionals report having education in nutrition. The difference is low-moderate in size (see Table 1).

Comparing the two groups on the frequency of specific types of formal education, results again indicated that nutrition professionals had more courses in mental health than mental health professionals had in nutrition. The difference between the two groups is only statistically significant in the share of people who had more than one graduate course.

When asked, “If you had formal training and resources, would you incorporate nutritional psychology into your practice?”, of those respondents (*n* = 196), the vast majority (92.3%; *n* = 181) answered that they would or that they already do, while 6.1% (*n* = 12) responded with “Maybe”. Only 1 participant gave a clear “No”, while 2 stated that they are retired. These findings point to a strong professional interest and a pressing need for formal training pathways. All participants answered this question.

Regarding informal training in nutrition related to mental health or mental health related to nutrition, all nutrition professionals who answered the question indicated that they had at least some informal training. A total of 92.7% (*n* = 76) of all participating mental health professionals reported having some form of informal training in nutrition related to mental health. Specifically, 66.3% (*n* = 61) of all participating mental health professionals or 74.4% of nutrition professionals who answered the question and 72.3% (*n* = 47) of all participating nutrition professionals or 90.4% of nutrition professionals who answered the question indicated being self-taught through resources such as books, research, or webinars, either alone or in combination with other forms of informal training. A total of 56.5% (*n* = 52) of all participating mental health professionals or 63.4% of those who answered the question and 67.7% of all participating nutrition professionals (*n* = 44) or 84.6% of nutrition professionals who answered the question reported participation in non-degree workshops, online courses, or webinars. Informal mentoring or on-the-job professional experience was reported by 31.5% (*n* = 29) of all participating mental health professionals, which is 35.4% of the mental health professionals who answered the question, and by 41.5% (*n* = 27) of all participating nutrition professionals or 51.9% of nutrition professionals who answered the question. These findings suggest that, in the absence of structured academic pathways, many professionals turn to informal, self-guided, or experiential routes to acquire nutrition-related knowledge.

Comparing mental health and nutrition professionals on the types and quantity of informal training related to mental health, the results again indicate that nutrition professionals report more informal training in mental health than mental health professionals report having in nutrition. Cramer’s V values are low, but one of the three crosses the 0.05 statistical significance threshold (see Table 1). Comparing the overall number of informal training types reported (informal training quantity), the results of the U test show a statistically significant difference of low-to-moderate magnitude in favor of nutrition professionals (see Table 1).

### 3.3. Perceived Barriers to Integrating Nutrition into Mental Healthcare

Respondents were asked to rate six predefined potential barriers to the integration of nutrition into mental healthcare on a scale from 1 (No barrier) to 5 (A very large barrier).

The most frequently cited barrier was the “Lack of training”, which received the highest average importance rating (M = 3.73, SD = 1.10). The “Unclear professional boundaries” also emerged as a major obstacle (M = 3.16, SD = 1.16). Similarly, the “Lack of a common language” (M = 3.10, SD = 1.15) was considered highly relevant by respondents.

The “Limited access to resources” was perceived as somewhat less important (M = 2.91, SD = 1.25), followed by “Time constraints in sessions” (M = 2.85, SD = 1.29).

In contrast, the “Lack of evidence” was perceived as the least critical barrier (M = 2.03, SD = 1.12).

To examine the associations among participants’ ratings of specific barriers, an exploratory factor analysis was conducted (Table 2).

When comparing mental health and nutrition professionals’ ratings of specific barriers, the results showed no statistically significant differences (see Table 3).

Within this sample, lack of training received the highest mean barrier rating, whereas lack of evidence received the lowest. These ratings reflect respondents’ perceptions of the listed barriers.

### 3.4. Ethical and Professional Concerns Related to Integration

The most highly rated concerns were “Ethical or liability concerns” (M = 2.68, SD = 1.29) and “Risk of working outside my professional scope” (M = 2.64, SD = 1.33), reflecting a broad awareness of ethical, legal, and professional boundaries when incorporating nutritional components into psychological care. Somewhat lower ratings were assigned to “Lack of confidence in nutrition knowledge” (M = 2.31, SD = 1.25), suggesting that many professionals feel underprepared to discuss or apply nutritional concepts in clinical settings, and to “Fear of confusing and overwhelming clients” (M = 2.31, SD = 1.15). “Billing concerns” were perceived as the least important concern (M = 2.25, SD = 1.32).

### 3.5. Education in Nutrition Within Mental Health, Barriers, and Discussing Nutrition/Mental Health with Patients

When exploring correlations between the quantity of formal and informal education about nutrition in mental health with perceptions of barriers and frequency of discussing mental health (by nutrition professionals) or nutrition (by mental health professionals), results generally indicated that nutrition professionals with more informal training (see Table 4) about nutrition in mental health reported discussing mental health with their patients more often. Similarly, mental health professionals with more formal training in nutrition tended to report discussing nutrition with their patients more frequently.

Perceptions of barriers were not associated with the quantity of either formal or informal training in the complementary area.

### 3.6. Perceptions and Experiences of Interprofessional Collaboration

This section distinguishes between professionals’ perceptions, their attitudes and beliefs about the value of collaboration, and their experiences (i.e., their actual involvement in collaborative activities). When asked whether interprofessional collaboration between mental health and nutrition professionals would improve patient outcomes, the vast majority of respondents expressed a strongly positive view. Specifically, among the participants who answered this question (*n* = 133), 72.9% (*n* = 97) strongly agreed, and 23.3% (*n* = 31) agreed. Only 2.3% (*n* = 3) were neutral, and just 2 participants (1.6%) expressed disagreement or strong disagreement. A total of 63 participants did not answer this questions.

Among respondents who answered this item, agreement with the perceived potential value of collaboration was high.

Despite this clear consensus on the potential benefits of collaboration, actual experience in collaborative practice was more varied. While 10.9% (*n* = 10) of mental health professionals reported collaborating with a dietitian or nutritionist regularly, 37% (*n* = 34) reported doing so occasionally, and somewhat less than half of mental health professionals (41.3%; *n* = 38) had never collaborated in patient care or were not sure whether they have. A total of 10.9% (*n* = 10) of mental health professionals did not answer this question.

Similarly, 36.9% of nutrition professionals (*n* = 24) reported regularly cooperating with a psychologist or other mental health professionals, while 12.3% (*n* = 8) reported doing so occasionally. A total of 26.2% (*n* = 17) of nutrition professionals reported never having cooperated with a psychologist or other mental health professional or being unsure whether they have.

A total of 7 mental health professionals and 16 nutrition professionals did not answer the question about collaborative experiences.

These results highlight a strong perceived value of interdisciplinary collaboration and a clear interest in expanding such partnerships, although current opportunities for direct collaborative practice remain limited for many professionals.

### 3.7. Sensitivity Analyses

The analyses presented in this paper include one aggregation of items (the quantity of formal training) created using a priori weights based on our judgement and another aggregation—factor score that is primarily interpreted based on a single item with high loading and another item with a moderate loading—factor 2—resource barriers. As these are essentially provisional exploratory aggregations of items, rather than well-established scores/latent constructs, we conducted sensitivity analyses to explore whether our conclusions would be affected if the quantity of formal training was calculated as an unweighted composite, i.e., if it was simply a count of the types of formal education a participant selected in their answer and when resource barriers were simply the sum of the two items primarily loading on factor 2, i.e., time constraint in sessions and limited access to nutrition resources.

The results of these analyses showed that the correlation between the number of formal education types a participant selected and the formal education quantity variable presented in this manuscript is 0.939. The Cramer’s V coefficient for the association between whether a participant is a mental health or nutrition professional and the number of formal education types listed is 0.336 (*p* = 0.002). The correlation between the number of formal education types selected and the frequency of discussing nutrition in clinical practice is 0.497, while the correlation with discussing mental health among nutrition professionals is 0.010. The correlations with remaining variables presented in Table 4 are all close to 0 and statistically nonsignificant We conclude that our conclusions presented in this paper regarding the quantity of formal education in the complementary field are not sensitive to whether the quantity of formal education is expressed as the count of types of formal education selected or an a priori-weighted composite like the one used in this paper.

If resource barriers are expressed as a sum of the two items with the highest loading on factor 2 (presented in Table 2), the correlation between that score and the factor 2 factor score is 0.888. The difference in central tendencies between mental health and nutrition professionals on this score is statistically nonsignificant (Mann–Whitney U = 2767, z = −0.802, *p* = 0.422). Correlations between this factor score and the quantity of formal and informal education scores are also close to 0 and statistically nonsignificant. We conclude that the conclusions presented in this manuscript are not sensitive to whether resource barriers are expressed as a factor 2 score or as an unweighted composite of items Time constraint in sessions and Limited access to nutrition resources, from the list of potential barriers.

## 4. Discussion

This exploratory study examined the current landscape of interdisciplinary integration between mental health and nutrition professions by analyzing self-reported training, clinical practices, perceived barriers, and interest in collaborative care models. Within this sample, many respondents reported interest in bridging psychological and nutritional sciences, limited formal training in the complementary field, and relatively infrequent interprofessional collaboration. These results align with the American Psychological Association’s growing attention to the bidirectional interplay among dietary intake, psychological processes, and mental health, with nutritional psychology emerging as an interdisciplinary field within the psychological sciences at the interface of nutrition and mental health [27]. Within this context, nutritional psychology should be distinguished from several adjacent but related domains. Consistent with recent conceptual frameworks, nutritional psychology is a psychologically grounded, interdisciplinary field situated within the broader psychological sciences that focuses on the cognitive, emotional, behavioral, sensory–perceptual, interoceptive, environmental, and psychosocial processes underlying eating behavior and psychological functioning, as well as the reciprocal relationships through which nutrition and psychological processes influence one another across normative, subclinical, and clinical contexts [13]. In contrast, nutritional psychiatry is an evidence-based, transdisciplinary field concerned with understanding, preventing, and managing psychiatric symptoms and clinically relevant mental health outcomes through nutrition-related exposures and interventions [28,29]. Although both fields examine the relationship between nutrition and mental health and may investigate overlapping phenomena, they are distinguished primarily by their explanatory orientation and explanatory targets rather than by the specific topics they study. Nutritional Psychology should also be distinguished from nutrition counselling and interdisciplinary models of care, despite areas of overlap in their application and objectives.

A central finding concerns the lack of formal training in nutritional psychology across professional groups. The extent of this lack was similar among groups of mental health professionals and nutrition professionals. Within this sample, almost half of the respondents who reported their formal education in the complementary field reported no formal education in the complementary field, and the most common form of training consisted of non-degree certificates or workshops. While only a minority had completed graduate-level coursework or comprehensive academic programs, a large proportion reported engaging in self-directed learning, such as reading, online webinars, and informal mentoring. This finding may indicate a strong intrinsic motivation to acquire relevant knowledge. These findings are consistent with previous studies [14,17] that have identified a persistent gap between professional interest in nutritional psychology training and institutional support. Results also show a correlation between the quantity of training in the complementary field and the frequency with which they discuss it in their practice. The more training participants had, the more likely they were to discuss the topics from the complementary field. Interestingly, this association between how often they discuss topics from the complementary field and the quantity of training was statistically significant with informal training for nutrition professionals and with formal training for mental health professionals, while not being statistically significant for the other type of training. It is important to note that the relationships established in this study are correlational and cross-sectional, and therefore cannot support causal inferences.

Despite the lack of formal preparation, most psychologists and other mental health professionals who responded reported addressing nutrition at least occasionally in clinical sessions, and nearly all nutrition professionals who responded reported discussing mental health at least occasionally. This within-sample contrast may identify an implementation issue worthy of further investigation. However, the fact that many engage in cross-disciplinary discussions without formal training also raises ethical and professional considerations regarding boundaries of competence. This association indicates that greater self-reported educational exposure was related to more frequent self-reported discussion of topics from the complementary field. The coexistence, within this sample, of frequent cross-disciplinary discussions and limited reported formal training raises questions for future research concerning competence, professional boundaries, and the availability of collaborative frameworks.

Analysis showed that these barriers can be grouped into professional barriers and resource barriers. Nutrition professionals and mental health professionals perceived these barriers as similarly important. Although mean ratings showed minor numerical differences across some barriers, none of the between-group comparisons reached statistical significance; therefore, these trends should not be overinterpreted. It should be noted that because the “Factor score—Resource barriers” is primarily based on a single item with high loadings and one more moderate item, the results reported that involve this factor should be treated as a secondary analysis.

While nearly all participants who responded to the question believed that collaboration between mental health and nutrition professionals could improve patient outcomes, fewer than half reported any past collaborative experience, and the share of those who reported engaging in regular interprofessional care was even smaller. This striking discrepancy between perceived value and actual implementation underscores the need for institutional support, defined pathways, and accessible models of collaboration. Respondents’ agreement that collaboration could improve patient outcomes reflects an attitude or expectation. Because the study collected no measures of quality of care, clinical effectiveness, or patient-level outcomes, it provides no evidence that collaboration actually improves these outcomes.

Taken together, the closed-ended survey responses indicate that respondents rated lack of training, unclear professional boundaries, and lack of a common language as salient barriers and generally expressed positive attitudes toward interdisciplinary collaboration. These findings may help generate hypotheses and inform the future development of interdisciplinary initiatives, but they do not establish the effectiveness, necessity, or feasibility of any specific educational, clinical, organizational, or policy intervention. Beyond these empirical findings, potential directions for further investigation may include structured interdisciplinary curricula, joint professional guidance, supervision and referral frameworks, and organizational or reimbursement mechanisms intended to support collaborative care. However, the present study did not evaluate these approaches, their sustainability, or their effects on professional practice and patient outcomes. They should therefore be regarded as proposals requiring stakeholder consultation, prospective evaluation, and patient-level research rather than as direct evidence-based recommendations arising from this survey.

## 5. Limitations

This study has several limitations. First, the data was based on self-reports, which introduces the possibility of recall bias and social desirability effects. Recruitment was conducted through professional and institutional networks associated with the CNP and collaborating institutions. Because CNP provides training in nutritional psychology, this recruitment pathway may have preferentially reached individuals already familiar with or interested in the field and related training. The sample may therefore overrepresent cross-disciplinary engagement, favorable attitudes toward collaboration, and interest in further education. These generalizability of the findings to the broader populations of mental health and nutrition professionals may therefore be limited, and the magnitude of this potential selection bias cannot be determined from the available data. Furthermore, professional status of study participants could not be independently verified, and geographical information was not collected, limiting the characterization of the sample. Moreover, because the number of individuals reached or eligible was unknown, an overall response rate could not be calculated. Therefore, the results should be interpreted as likely reflecting a motivated subgroup of professionals rather than the broader population of mental health and nutrition practitioners. Moreover, some items had substantial non-response rates; therefore, percentages should be interpreted with attention to the item-specific denominator. The second factor derived from factor analysis of barrier ratings is defined primarily by one item with a high loading and one with a moderate loading. Because of this, it should primarily be treated as a convenient interpretative aggregation of the examined barriers rather than a well-established latent trait. The original survey did not collect information on participants’ years of professional experience, geographic location, licensure status, practice setting, or patient populations served. Consequently, the professional and contextual characteristics of the sample could not be adequately described, eligibility could not be independently verified, and subgroup analyses could not be conducted. It therefore remains unknown at this point whether the reported practices, perceived barriers, and attitudes toward interdisciplinary collaboration vary across career stages, regulatory contexts, healthcare systems, clinical settings, or patient populations. Future surveys should systematically collect these variables to support eligibility verification, stratified analyses, and a more robust assessment of the generalizability of the findings. Importantly, the study did not objectively assess professional knowledge, competence, or clinical behaviour. Neither participation in training nor the reported frequency of discussing topics from the complementary field can be assumed to reflect the accuracy, appropriateness, or ethical quality of professional practice. The study also did not assess whether these practices influenced the quality of care or patient outcomes. Future research should combine self-reported data with standardized knowledge assessments, case-based or competency-based evaluations, direct or simulated observations of clinical practice, and patient-level outcome measures. Such approaches would help determine whether interdisciplinary training translates into appropriate professional behavior and clinically meaningful benefits. Also, the survey did not provide explicit operational definitions of concepts such as “addressing nutrition”, “discussing mental health”, “nutritional advice”, and “incorporating nutritional psychology”. While we believe that these concepts are self-explanatory, we do recognize that a lack of operational definitions might have resulted in some differences between participants in which these concepts were understood. Finally, the cross-sectional design does not allow for causal inferences regarding the impact of training or collaboration on clinical outcomes. Taken together, these limitations substantially restrict the interpretation and generalizability of the findings.

Despite this, the study offers an exploratory overview of current attitudes, experiences, and perceived needs among professionals working at the intersection of nutrition and mental health. The results highlight not only a substantial readiness for interdisciplinary integration, but also concrete educational, systemic, and regulatory gaps that must be addressed to translate interest into effective clinical practice. Furthermore, future research should include validated knowledge tests, observed practice measures, and patient-level outcomes to substantiate these findings.

## 6. Conclusions

This study provides an exploratory overview of how mental health and nutrition professionals participating in our study perceive, implement, and envision interdisciplinary collaboration. The findings reveal a clear mismatch between strong professional interest in integration and the limited availability of formal training and structured collaborative opportunities. Some respondents reported discussing topics from the complementary field or previous collaborative experience, while limited formal training and concerns regarding professional boundaries were also reported. Among respondents who answered the relevant items, there was strong support for the potential value of structured, evidence-informed, and ethically grounded educational frameworks and collaborative models. Their responses highlight interest in developing core competencies and adaptable curricula that can evolve alongside emerging scientific evidence and support safe interdisciplinary practice.

That said, this was primarily an exploratory study. Future research and practice-development initiatives could examine interdisciplinary curricula, professional scopes of practice, accreditation mechanisms and institutional approaches supporting team-based care. Prospective evaluation of their feasibility, implementation, sustainability, effects on professional practice, and association with patient-level outcomes will help determine how nutritional and psychological care can be integrated effectively in clinical settings.

## Figures and Tables

**Table 1:** Formal and informal training of mental health professionals and nutrition professionals in the complementary area, percentages relative to the total number of participants.

**Education/Training Variable**	**Mental Health Professionals**	**Nutrition** **Professionals**	**Cramer’s V**	**Holm–Bonferroni Corrected Statistical Significance**	
**Formal training**					
One undergraduate course (e.g., a 101 course)	4.3%	12.32%	0.178	0.1600	
Professional certificate/training program	25.0%	33.8%	0.143	0.196	
More than one undergraduate course	5.4%	23.1%	0.310	0.025	
Graduate coursework	7.6%	10.8%	0.077	0.377	
**Informal training**					
Self-taught	66.3%	72.3%	0.197	0.115	
Participation in workshops, online courses, or webinars (non-degree)	56.5%	67.7%	0.229	0.048	
Informal mentoring or professional experience	31.5%	41.5%	0.164	0.174	
**Education/Training Variable**	**Mental Health Professionals**	**Nutrition** **Professionals**	**Mann–Whitney U (z)**	**Statistical** **Significance**	**r_r_bis**
Quantity of formal training score, M (SD)	1.148 (1.726)	2.404 (2.411)	1381.5 (−3.559)	*p* < 0.001	0.344
Quantity of informal training score, M (SD)	1.732 (0.917)	2.269 (0.770)	1444 (−3.306)	*p* < 0.001	0.323

Note. Scores of formal and informal training quantities are not comparable. Statistical significance statistics in the rows representing formal and informal training modes were obtained from Chi-square tests of independence, with Cramer’s V reported as an associated effect size measure. These statistical significance statistics were adjusted for multiple comparisons using the Holm–Bonferroni procedure for a family of 7 comparisons. Statistical significance statistics in the bottom two rows are statistical significance levels of the Mann–Whitney U tests.

**Table 2:** Loadings and percentages of variance explained by rotated factors extracted from participants’ responses about barriers for integrating nutrition into mental health varimax rotation was applied, and principal axis factoring.

Barrier Item	Professional Barriers (Factor 1)	Resource Barriers (Factor 2)	Uniqueness
Percentage of variance explained by the factors	26.60%	21.73%	
Lack of evidence	0.309		0.832
Lack of training	0.729		0.386
Lack of common language	0.722	0.320	0.376
Unclear professional boundaries	0.561		0.655
Time constraint in sessions		0.472	0.700
Limited access to nutrition resources		0.891	0.151

Inspection of factor loadings indicates that participants seem to group ratings into two latent dimensions: (1) professional barriers, represented primarily by unclear professional boundaries, lack of common language and training, and (2) resource barriers, primarily represented by limited access to nutrition resources and time constraints in sessions. Note: Bartlett’s test of sphericity Chi Square= 297.50, df = 15, *p* < 0.001. The overall KMO measure of sampling adequacy was 0.799. Individual measures of sampling adequacy ranged from 0.773 to 0.885. The decision to keep two factors was based on the results of Horn’s parallel analysis.

**Table 3:** Barrier ratings and the comparison of average ratings assigned to different barriers by nutrition and mental health professionals.

Barrier	Mental Health Professionals, M (SD)	Nutrition Professionals, M (SD)	Mann–Whitney z	*p*	r_r_bis
Lack of evidence	2.000 (1.079)	2.015 (1.152)	−0.075	0.941	−0.007
Lack of training	3.728 (1.017)	3.769 (1.129)	−0.451	0.653	0.040
Lack of common language	3.087 (1.164)	3.046 (1.138)	−0.378	0.707	−0.034
Unclear professional boundaries	3.13 (1.206)	3.308 (1.089)	−0.825	0.410	0.075
Time constraint in sessions	2.641 (1.297)	2.892 (1.312)	−1.233	0.218	0.113
Limited access to nutrition resources	2.859 (1.163)	2.908 (1.366)	−0.091	0.929	0.008
Factor score—Professional barriers	0.004 (0.948)	0.028 (1.001)	−0.255	0.799	0.024
Factor score—Resource barriers	−0.068 (0.954)	−0.006 (1.105)	−0.308	0.759	0.029

Note. Because the “Factor score—Resource barriers” is primarily based on a single item with high loadings and one more moderate item, the results reported here that involve this factor should be treated as a secondary analysis.

**Table 4:** Spearman’s correlation between the quantity of formal and informal education in nutrition and mental health, the frequency of discussing mental health or nutrition with clients, and perceptions of barriers.

Spearman’s Rank Correlations	Quantity of Informal Education	Quantity of Formal Education
Statistics Calculated	Correlation; 95% Confidence Interval (Unadjusted *p* Value; Adjusted *p* Value)	Correlation; 95% Confidence Interval (Unadjusted *p* Value; Adjusted *p* Value)
Nutrition professionals’ frequency of discussing mental health in practice (nutrition professionals only, *n* = 52)	0.314; 0.045–0.541 (0.023; 0.046) *	0.027; −0.248–0.298 (0.850; 0.850)
Mental health professionals’ frequency of discussing nutrition in practice (mental health professionals only, *n* = 82)	0.206; −0.012–0.405 (0.063; 0.063)	0.479; 0.292–0.63 (*p* < 0.001; *p* < 0.001) *
Lack of evidence (combined mental health and nutrition professional sample)	−0.016; −0.185–0.154 (0.853; 1.000)	−0.002;−0.172–0.168 (0.978; 1.00)
Lack of training (combined mental health and nutrition professional sample)	−0.015; −0.184–0.155 (0.868; 1.00)	0.008; −0.162–0.178 (0.923; 1.00)
Lack of common language (combined mental health and nutrition professional sample)	−0.119; −0.283–0.052 (0.171; 1.00)	−0.088; −0.254–0.083 (0.314; 1.00)
Unclear professional boundaries (combined mental health and nutrition professional sample)	0.011;−0.159–0.180 (0.899; 1.00)	0.058; −0.113–0.226 (0.505; 1.00)
Time constraint in sessions (combined mental health and nutrition professional sample)	−0.058; −0.225–0.113 (0.508; 1.00)	−0.052; −0.220–0.119 (0.551; 1.00)
Limited access to nutrition resources (combined mental health and nutrition professional sample)	−0.027; −0.196–0.143 (0.759; 1.00)	−0.108;−0.273–0.063 (0.218; 1.00)
Factor score—Professional barriers (combined mental health and nutrition professional sample)	−0.059; −0.226–0.112 (0.499; 1.00)	0.020; −0.151–0.190 (0.818; 1.00)
Factor score—Resource barriers (combined mental health and nutrition professional sample)	−0.017; −0.186–0.153 (0.844; 1.00)	−0.113; −0.278–0.058 (0.197; 1.00)

Note. * *p* < 0.05, The combined mental health and nutrition professional sample is 133 participants from correlations with formal education quantity and 134 for correlations with informal education quantity. Participants classified as “Other” were not included in these correlations as they did not report data on the quantity of formal or informal education in the complementary field. Unadjusted *p* values are *p* values without corrections for multiple comparisons. Adjusted *p* values are *p* values after Holm–Bonferroni correction for multiple comparisons. Correlations on the subgroup of mental health professionals have been corrected for a family of 2, correlations on the subgroup of nutrition professionals for a family of 2 as well, while correlations on the full sample were Holm–Bonferroni corrected for a family of 16. Confidence intervals are in the format lower boundary-upper boundary. Because the “Factor score—Resource barriers” is primarily based on a single item with high loadings and one more moderate item, the results reported here that involve this factor should be treated as a secondary analysis.

## Data Availability

The data presented in this study are available on request from the corresponding author due to privacy restrictions, raw survey responses cannot be publicly shared.

## Abbreviations

The following abbreviations are used in this manuscript:

ACT	Acceptance and Commitment Therapy
APA	American Psychological Association
BMI	Body Mass Index
CBT	Cognitive Behavioral Therapy
CBT-IE	Interprofessional Enhanced Cognitive Behavioral Therapy
CFR	Code of Federal Regulations
CNP	Center for Nutritional Psychology
HHS	Health and Human Services
IBM	International Business Machines
IFEDD	International Federation of Eating Disorder Dietitians
IRB	Institutional Review Board
JASP	Jeffreys’s Amazing Statistics Program
MI	Motivational Interviewing
SPSS	Statistical Package for the Social Sciences
